# Ictal Burst Suppression: A Case Report and Brief Overview

**DOI:** 10.7759/cureus.106222

**Published:** 2026-03-31

**Authors:** Bertrand Liang, Lauren Ilagan, Mariam Akinwale, Scott Lambert

**Affiliations:** 1 Department of Neurology, University of Colorado School of Medicine, Colorado Springs, USA; 2 Department of Neurology, St. Joseph's Medical Center, Stockton, USA; 3 Department of Psychiatry, St. Joseph's Medical Center, Stockton, USA

**Keywords:** anoxia, burst suppression, cardiac arrest outcome, ictal burst suppression, neurologic prognosis, seizures

## Abstract

In patients suffering catastrophic injury to the nervous system, a burst suppression pattern found on EEG is a rare finding. Usually, this pattern consists of bursts of mixed-frequency, variable-morphology waves alternating with suppressed periods and attenuation to an undetectable signal. This pattern can be difficult to assess, as burst suppression may be associated either with limited brain activity or with both clinical and electrographic seizures (ictal burst suppression). While this can occur at all ages, in adults it is most often seen with significant anoxic insult. We report a case of anoxic ictal burst suppression in a patient after cardiac arrest that was initially thought to represent anoxic myoclonus. Correlative clinical facial twitching and arm tonicity and flexion, which were abrogated by benzodiazepine and anesthetic administration, confirmed the ictal activity. Ictal burst suppression can be challenging both to recognize and to treat. We briefly review this phenomenon and the hypotheses regarding its potential mechanisms.

## Introduction

Anoxic ictal burst suppression is a rare and complex EEG pattern observed in patients following cardiac arrest and other severe hypoxic-ischemic brain injuries [1]. This pattern represents a paradoxical phenomenon in which rhythmic epileptiform discharges emerge in the context of severe anoxic brain injury, typically manifesting as a burst-suppression pattern with ictal characteristics. Recognition of burst suppression as an ictal phenomenon carries direct implications for treatment urgency, as aggressive seizure management may be warranted, and for goals-of-care discussions, as this pattern is associated with severe brain injury and poor prognosis. The recognition and understanding of this pattern have important implications for prognosis, treatment decisions, and determination of brain death criteria [2]. A key diagnostic challenge lies in distinguishing ictal burst suppression from anoxic myoclonus, which may present with similar clinical manifestations but represents a fundamentally different pathophysiology and does not respond to antiseizure treatment.

The exact mechanisms underlying anoxic ictal burst suppression remain incompletely understood, but several pathophysiological factors likely contribute to its development. Following cardiac arrest, the brain undergoes a cascade of injurious processes, including energy failure, excitotoxicity, oxidative stress, and inflammation. These processes can lead to widespread neuronal damage while paradoxically creating conditions for abnormal synchronous neuronal firing [3].

The burst-suppression pattern itself reflects a profound disturbance in cerebral metabolism and neuronal function. The “bursts” represent periods of synchronized neuronal activity, while the “suppression” periods reflect profound cortical inactivity. When these bursts take on ictal characteristics, such as rhythmic, evolving patterns with stereotyped morphology, the pattern is designated as ictal burst suppression [3-6].

The ictal component may arise from several mechanisms. Damaged neurons may exhibit increased membrane instability and altered ion channel function, predisposing them to spontaneous depolarizations [1]. Additionally, impaired inhibitory neurotransmission and disrupted cortical-subcortical networks may facilitate the emergence of epileptiform activity. Severe metabolic compromise prevents normal termination mechanisms for seizure activity, resulting in persistent ictal-appearing bursts [5,6].

We present a case of ictal burst suppression in a patient who was initially thought to have anoxic myoclonus but was later found to have tonic seizures and status epilepticus.

## Case presentation

An 86-year-old male with a history of Parkinson disease, type 2 diabetes, hypertension, congestive heart failure, restless leg syndrome, prostatic hypertrophy, and acute-on-chronic kidney disease presented with altered mental status to an outside hospital. He had recently been hospitalized for a urinary tract infection and had been discharged to a skilled nursing facility (SNF) one week earlier; he presented again to the outside ED from the SNF with significantly worsening leukocytosis and altered mentation. He was transferred to our medical center for further evaluation and a higher level of care because there was concern for acute leukemia. Subsequent hematologic evaluation, including peripheral smear review, was not consistent with acute leukemia; the leukocytosis was attributed to a severe infectious/inflammatory process.

In the ED, vital signs revealed a blood pressure of 144/81 mmHg, heart rate of 115 beats per minute (bpm), respiratory rate of 22 breaths per minute, oxygen saturation of 95% on room air, and an elevated temperature of 37.9 degrees Celsius. Pertinent laboratory evaluation revealed a WBC count of 34 thousand/mm3, hemoglobin of 11.4 g/dL, hematocrit of 33%, platelets of 445 thousand/mm3, INR of 1.2, sodium of 129 mmol/L, B-type natriuretic peptide of 822 pg/mL, troponin of 0.79 ng/mL, and procalcitonin of 0.23 ng/mL. Urinalysis showed 1-2 WBCs (Table 1).

**Table 1:** Laboratory values at presentation to the ED.

Parameter	Reference values	Results
Sodium	136-145 mmol/L	129
Potassium	3.5-5.1 mmol/L	4
Chloride	96-106 mmol/L	103
CO_^2^_	20-30 mmol/L	24
Blood urea nitrogen	6-24 mg/dL	28
Creatinine	0.65-1.36 mg/dL	1.9
Glucose	70-99 mg/dL	134
Hemoglobin	12.0-17.5 g/dL	11.4
Hematocrit	41-50%	33
Platelets	150,000-450,000/µL	445,000
WBC count	3.7-11.8 × 10³/µL	34
B-type natriuretic peptide	Less than 100 pg/mL	822
Troponin	Less than 0.04 ng/mL	0.79
Procalcitonin	Less than 0.1 ng/mL	0.23
Urine WBC	0-5/hpf	1-2
International normalized ratio	0.8-1.2	1.2

The patient was admitted to the hospital. While waiting for a bed, he suffered a cardiac arrest and required 13 minutes of resuscitation to achieve return of spontaneous circulation. He was intubated and transferred to the intensive care unit, where examination showed a lack of response to verbal or tactile stimuli, a temperature of 36.9 degrees Celsius, a heart rate of 60 bpm, and a blood pressure of 113/61 mmHg. He was noted to have “myoclonus,” described as movements of his facial musculature bilaterally. There was also concern for myoclonic movements of the left upper extremity. Targeted temperature management (TTM) was not initiated following cardiac arrest given the patient’s overall clinical trajectory, advanced age, and significant comorbidities.

Neurologic consultation was obtained. Neurologic examination revealed no response to voice, tactile, or noxious stimuli. The pupils were bilaterally equal, round, 3 mm in diameter, and reactive to light with hippus. Dysconjugate gaze was noted, and corneal reflexes were present bilaterally, as was an oculocephalic reflex. The gag reflex to bronchial suctioning was absent, as was the cough reflex. No withdrawal to painful stimuli was present centrally or in the limbs. During the examination, the patient was noted to have episodes of rigidity and flexion of the left upper extremity, with left facial twitching. After approximately 1 minute, the patient’s left arm was no longer flexed and had diminished resistance to passive manipulation. This pattern then repeated every few seconds. Two mg of IV lorazepam was administered, which fully stopped the twitching and rigidity within 30 seconds.

**Figure 1:**
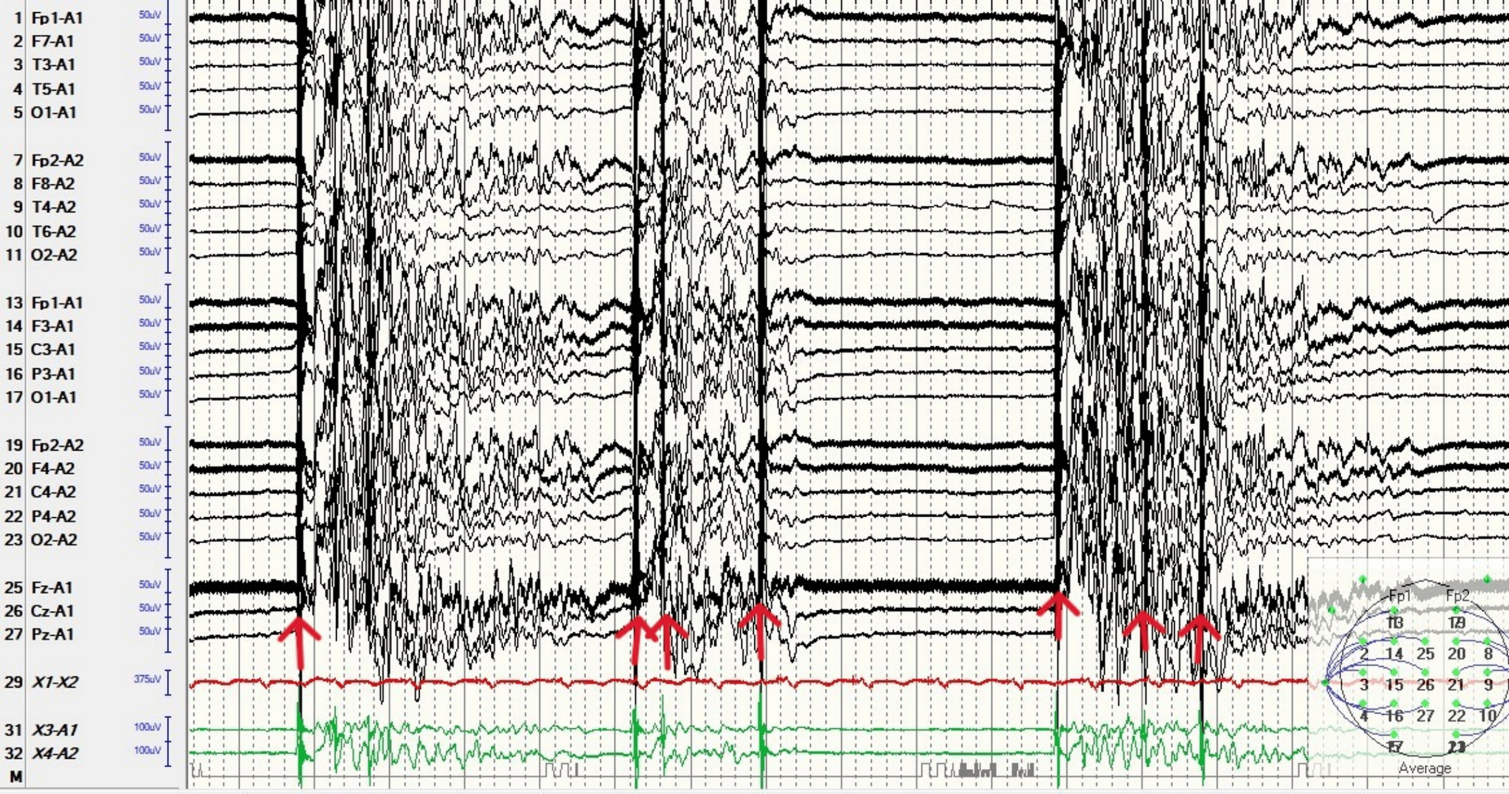
Ictal burst suppression pattern on EEG. Ictal burst suppression pattern in a patient with anoxic insult. Note the red arrows indicating muscle contraction, correlating clinically with facial twitching and left arm tonicity. EEG recording parameters: international 10-20 electrode array, longitudinal and transverse bipolar montages, low filter 1 Hz, high filter 70 Hz, with EKG electrodes.

**Figure 2:**
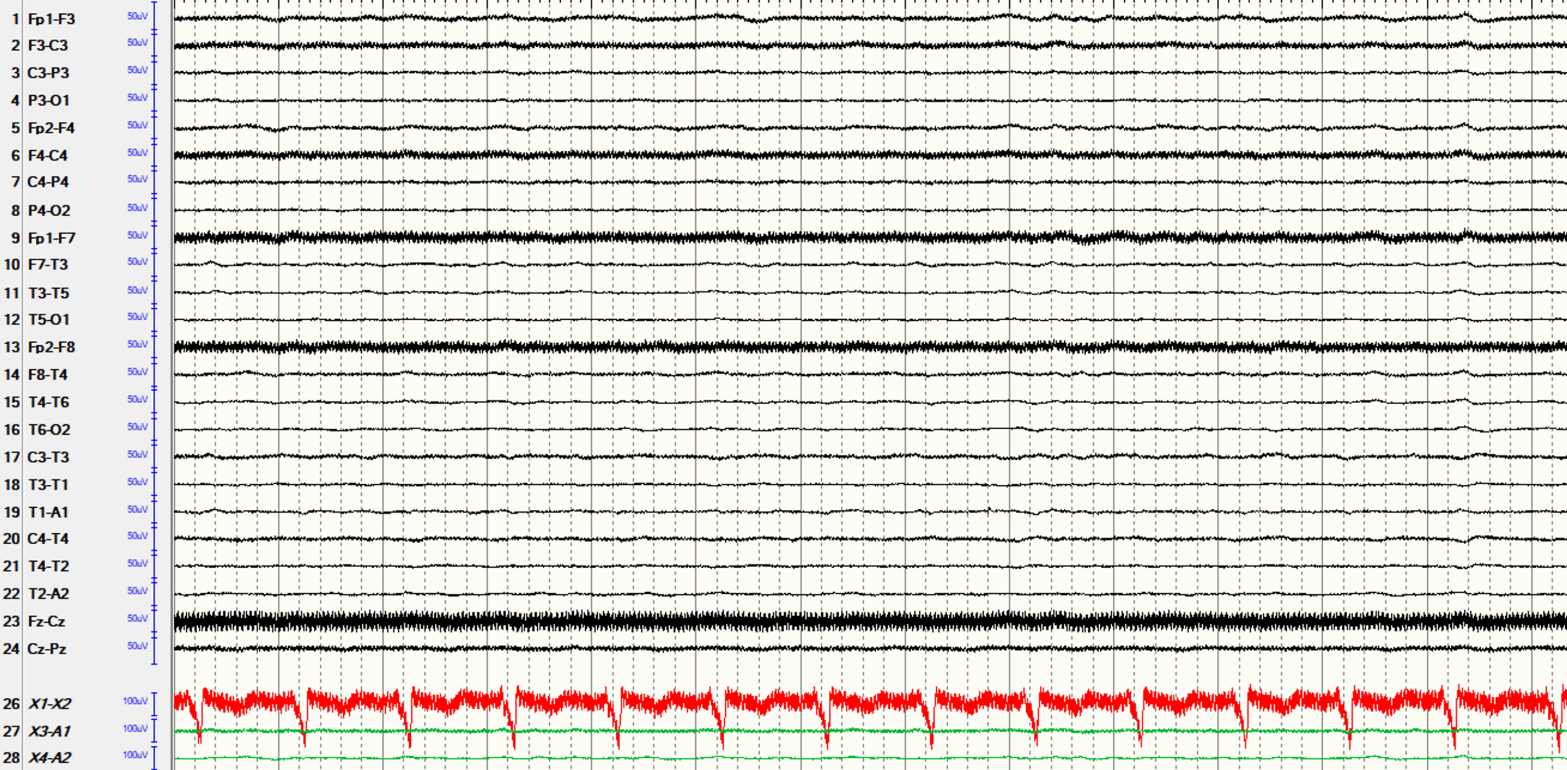
EEG pattern after treatment with propofol. EEG after treatment with propofol, showing overall background suppression with no clinical correlates. EEG recording parameters: international 10-20 electrode array, longitudinal and transverse bipolar montages, low filter 1 Hz, high filter 70 Hz, with EKG electrodes.

An EEG was obtained emergently (Figure 1). The recording utilized an international 10-20 electrode array with longitudinal and transverse bipolar montages, low filter settings of 1 Hz, and high filter settings of 70 Hz; the initial recording was one hour in duration. This revealed a burst-suppression pattern, with characteristic sharp waves accompanied by muscle artifact temporally correlated with burst activity, coincident with increased tonicity in the left upper extremity and facial twitching. Although lorazepam initially aborted the clinical seizure activity, ictal activity recurred within approximately 1 to 2 minutes. Levetiracetam was loaded (20 mg/kg) but did not achieve seizure control. Propofol was then initiated with a 2 mg/kg bolus, followed by a continuous infusion at 10 mg/kg/h for 10 minutes, then 8 mg/kg/h for 10 minutes, and then 6 mg/kg/h thereafter. This stopped both the ictal burst suppression and the tonicity and facial twitching (Figure 2). Longer-term EEG monitoring continued for three additional hours after initiation of propofol, confirming seizure suppression. The patient subsequently had difficulty maintaining blood pressure and required significant pressor support, which then began to fail. Advanced neuroimaging was not performed; given the patient’s acute hemodynamic instability, brain CT and MRI were deferred at the family’s request. The patient’s family opted for comfort care measures, and propofol was discontinued; the patient expired shortly thereafter.

## Discussion

Ictal burst suppression represents a diagnostic challenge due to its resemblance to other burst-suppression patterns that are not ictal in nature. The key to recognizing ictal burst suppression lies in demonstrating that the burst-suppression pattern itself represents ongoing seizure activity. This may be evidenced by clinical correlation with seizure semiology, response to antiseizure medications (either improvement or worsening of the pattern), evolution from or to other ictal patterns, or the presence of clinical manifestations that correlate with the bursts themselves [7].

The distinction between ictal burst suppression and other causes of burst suppression is both mechanistic and etiologic. Induced burst suppression occurring in the context of anesthesia or therapeutic hypothermia is generally reversible and does not carry the same dire prognostic implications. Similarly, postictal burst suppression following status epilepticus, while indicating severe seizure activity, represents a transitional state rather than ongoing seizure activity. In contrast, true ictal burst suppression suggests active, ongoing epileptic activity that is both refractory to treatment and associated with severe underlying brain pathology [8].

Previously reported cases of ictal burst suppression in adults encompass several underlying etiologies [5,7,9]. Ictal burst suppression has been described in the context of acute encephalitis, acute neurological deterioration in chronic epilepsy syndromes, and, particularly, catastrophic nervous system anoxia. In younger patients, metabolic etiologies associated with ictal burst suppression can include severe hypoglycemia, hyperammonemia, mitochondrial disorders, and certain organic acidemias. In such cases, ictal burst suppression may represent a manifestation of acute metabolic decompensation, and correction of the underlying metabolic derangement may lead to improvement in the EEG pattern, although long-term neurological sequelae are common [10]. In adults, the usual etiology is an anoxic event, often catastrophic in nature [2].

The electrographic features of ictal burst suppression vary across reported cases but share certain common characteristics. The bursts typically consist of high-amplitude (often exceeding 150-300 μV), sharply contoured, or spike-and-wave activity lasting 1-10 seconds. The suppression periods show either complete electrical silence or marked voltage attenuation (typically below 10-25 μV), lasting variable durations from less than one second to several seconds. The burst-to-suppression ratio varies, and in some cases, asymmetry between hemispheres has been noted [11].

Importantly, ictal burst suppression may evolve from other seizure patterns or may itself evolve into other patterns, including continuous epileptiform activity, extreme delta brush, or progressive electrical silence [5]. This evolution supports the ictal nature of the pattern. Additionally, some reports have documented clinical manifestations (such as myoclonic jerks, tonic stiffening, or autonomic changes) correlating temporally with the bursts [2,3]. In our case, there was initially some confusion, since the semiology of the patient’s symptoms was ascribed to presumed anoxic myoclonus rather than an ictal phenomenon. In fact, ictal burst suppression was diagnosed only after bedside clinical evaluation by the Neurology service, with subsequent corresponding EEG correlation. This illustrates the challenges of recognizing the clinical scenario of ictal burst suppression and the need for prompt recognition in order to treat expeditiously.

Management of ictal burst suppression can be difficult. The pattern is typically refractory to standard antiseizure medications, and even aggressive therapy often fails to achieve sustained seizure control [8]. The use of multiple anti-epileptic agents is often required. If anesthetic agents such as pentobarbital or propofol are used, given that these agents per se can induce a burst-suppression pattern, careful ongoing evaluation of the patient is required to distinguish ictal burst suppression from non-epileptic pharmacologic burst suppression. Some clinicians have advocated titrating anesthetic agents to achieve complete electrical suppression rather than burst suppression [12], although this approach carries its own risks, including prolonged recovery, hemodynamic instability, and the potential for anesthetic complications.

The prognosis associated with ictal burst suppression is poor across reported cases [13]. High rates of mortality, severe neurological disability, and refractory epilepsy characterize the outcomes in patients with this EEG pattern. The poor prognosis likely reflects both the severe underlying pathology that gives rise to ictal burst suppression and the direct effects of uncontrolled seizure activity on the injured brain. The duration and intensity of ictal burst suppression may correlate with outcome, although the rarity of the pattern limits robust prognostic data [13]. However, in reported adult patients, ictal burst suppression represents an electrographic marker of irreversible or progressive neurological injury associated with either death or significant debilitation.

The mechanisms underlying ictal burst suppression remain speculative but likely involve severe disruption of the normal excitatory-inhibitory balance and neuronal network function. Hypotheses include the “metabolic exhaustion” hypothesis, wherein neurons are capable of generating bursts of hypersynchronous activity but cannot sustain this activity due to energy depletion, leading to periods of suppression [14], and the “inhibitory override” hypothesis, which proposes that excessive GABAergic inhibition, possibly as an endogenous protective mechanism against excitotoxicity, interrupts ongoing seizure activity, resulting in the suppression periods [6].

More recently, network-level dysfunction has been proposed, suggesting that ictal burst suppression may represent a pathological attractor state in severely compromised neural networks [5,6,15]. In this model, the brain alternates between two unstable states, a hyperexcitable state producing the bursts and a hypoexcitable state producing the suppression, without achieving stable normal activity. This framework helps explain why ictal burst suppression is so refractory to treatment: standard antiseizure medications may modulate one state but fail to restore normal network dynamics.

## Conclusions

We report a case of ictal burst suppression that was initially thought to represent anoxic myoclonus in a patient suffering cardiac arrest. Ictal burst suppression represents a rare but clinically significant EEG pattern that serves as a marker of severe underlying pathology. In the published literature, this pattern portends a poor prognosis in the vast majority of reported cases, although we acknowledge that prognostic conclusions from our individual case are limited by the absence of multimodal prognostication data (such as serum biomarkers or advanced neuroimaging) and the early transition to comfort care. Recognition of this pattern requires careful clinical and electrographic correlation, as burst suppression can occur in multiple non-ictal contexts. The etiologies associated with ictal burst suppression, ranging from metabolic catastrophes to acute brain injury, necessitate comprehensive diagnostic evaluation when this pattern is encountered.

Management of ictal burst suppression remains challenging, with most cases proving refractory to standard and even aggressive antiseizure treatment. The uniformly poor outcomes reported across case series underscore the need for improved understanding of the pathophysiological mechanisms underlying this pattern and for the development of novel therapeutic strategies. While rare, ictal burst suppression demands recognition by clinicians interpreting EEG, particularly in the context of refractory seizures or encephalopathy, as its identification has important implications for prognosis and treatment decisions.
